# The Influence of Autonomy-Supportive Teaching on EFL Students’ Classroom Autonomy: An Experimental Intervention

**DOI:** 10.3389/fpsyg.2021.728657

**Published:** 2021-09-08

**Authors:** Fakieh Alrabai

**Affiliations:** Department of English, Faculty of Languages and Translation, King Khalid University, Abha, Saudi Arabia

**Keywords:** autonomy, choice, motivation, relatedness, metacognition, autonomy-support, self-determination theory

## Abstract

Based on the framework of self-determination theory (SDT), this two-wave longitudinal empirical investigation examined the actual practicality of certain strategies that have been theoretically acknowledged as having potential positive effect on English as a foreign language (EFL) learner’s autonomy. Strategies targeting learners’ self-determined learning in the classroom in terms of satisfying learner basic psychological needs (BPNs) of autonomy, competence, and relatedness as well as SDT key concepts, such as learner sense of choice, intrinsic motivation, control over learning, goals and needs, and metacognitive skills, were implemented in a treatment group for 12weeks. A classroom observation was used to evaluate teachers’ autonomy-supportive teaching and a student self-report measure, and an observation were used to assess learners’ autonomy. The findings derived out from analyses of variance, covariance and a hierarchical regression revealed that the experimental intervention led to statistically significant increased EFL autonomy for learners in the experimental group. Learner perceived choice, autonomy support, competence, and intrinsic motivation mediated the relationship between teacher autonomy-supportive teaching and learner autonomy; with perceived choice being the strongest predictor of learner autonomy. These findings acknowledge the vital role of teacher autonomy-supportive teaching in promoting EFL learner autonomy and recommend that, beside satisfying their BPNs, students should always be granted a larger space of freedom of choice, more control over learning, and more involvement in decision-making process.

## Introduction

The concept of “autonomous learning” has been popular in language education for decades. It is nonetheless necessary to point out that there is no single conclusive definition for this concept due to its multifaced and complex nature. Little (1991, p. 4) conceptualized autonomy as “a capacity for detachment, critical reflection, decision-making, and independent action.” In another definition, Benson (2001) perceived autonomy as the capacity to take charge of one’s own leaning based on his desire, ability, and degree of freedom. For Little (2020, p. 1), language learner autonomy denotes “a teaching/learning dynamic in which learners plan, implement, monitor, and evaluate their own learning.”

A large body of related literature (e.g., Little, 2007; Dickinson, 1995; Ushioda, 2006; Brown, 2007; Raya et al., 2020; and many others) recognized learner autonomy to be powerfully related to many other learner variables such as high motivation, willingness to communicate, self-efficacy determining the success of language learning, the capacity for detachment, critical reflection, decision-making, and independent learning.

Autonomy-supportive teaching, according to Reeve (2016), is the delivery of instruction through an interpersonal tone of understanding that appreciates, supports, and vitalizes students’ psychological needs. Past research (e.g., Cheon et al., 2016; Reeve et al., 2019) asserted that this kind of practice helps students to experience high need satisfaction and low need frustration and is usually associated with positive behaviors on the part of the learner such as higher mastery motivation, greater perceived competence, promoted creativity, greater engagement, enhanced well-being, higher desire for taking challenges, better academic performance, and more persistence. In this regard, Ikonen (2013) emphasized that the movement toward autonomy-supportive teaching in institutional settings has proven to fit surprisingly well. According to him, an institutional setting is an appropriate environment for promoting learner autonomy because the different aspects of learner autonomy as interdependence, cooperation, the technical skills, and autonomy-related willingness, can naturally be incorporated into foreign language teaching. So, given the right conditions, autonomous language learning is not only possible but also practical in an institutional setting. Ellis and Sinclair (1989) went in line with Ikonen’s claim assuming that, when carefully designed and implemented, formal teaching can promote learner autonomy.

In EFL contexts, an autonomy-supportive environment is to be established and maintained in order to promote EFL learner autonomy. Earlier research (e.g., Black and Deci, 2000; Chua, 2010; Esch, 2010; Chinpakdee, 2020) described autonomy-supportive teaching in language learning as the provision of circumstances for language learners that empower them to take charge of the whole or part of their language learning in settings, where EFL learners’ frame of reference is recognized, external incentives and threats are decreased, and controlling language is avoided. As to put autonomy-supportive teaching in EFL instruction into practice, Benson (2011) recommended that a *pedagogy for autonomy* is to be adopted. According to him, *pedagogy for autonomy* pertains to the approaches that aim at fostering autonomy in a language classroom context using *pedagogical strategies for autonomy*, which are the discrete procedures those pedagogies incorporate.

There have been many theoretically-grounded approaches to fostering learner autonomy in the foreign language class. One of such approaches is that of Benson (2011). He classified autonomy-supportive approaches as mainly falling into in-class and out-of-class strategies. The in-class approaches are those pertaining to the learner, learning environment, curriculum, and the teacher. The learner-based strategies concern learner training and learner development while the classroom-based ones relate to strategies aiming at granting learners the decision-making power in their everyday learning content and procedures. The curriculum-based strategies, on the other hand, are those in which learner control is extended to the curricular level. Finally, the teacher-based approaches are the ones that work on developing learner autonomy in light of teacher autonomy. The out-of-class strategies in this framework refers to resource-based and technology-based approaches.

Littlewood (1996) framework was another approach for developing EFL learner autonomy. In this framework, autonomy consists of three domains: autonomy as a communicator (autonomy on a task level), as a learner (autonomy on learning level), and as a person (autonomy on a personal level). According to this model, in order to be autonomous in any of the three domains, two components need to be present, namely, ability, and willingness, both of which can further be divided into two underlying components: ability into knowledge and skills, and willingness into motivation and confidence.

One of the most important frameworks for developing autonomy is Dam’s (2011). According to Benson (2011) and Little (2020), this is a classroom-based approach to the development of learner autonomy, since it mainly deals with day-to-day learning management. The principles of this model are choice (making independent choices about student own learning on multiple levels ranging from what activity to do to taking part in course design), willingness (making curricular demands and guidelines clear for the students, and structuring lessons transparently), teacher support (providing learners with whatever assistance and guidance they might need to learn independently), authenticity (encouraging authentic use of the target language), and evaluation (providing tools for reflection, assessment and reassessment, and using tools as logbooks, portfolios, and posters).

Lewis and Reinders (2008) was another autonomy-enhancement framework. According to Ikonen (2013), this model is primarily concerned with improving willingness to take responsibility over learning. The principles of this model include teacher support (providing a rationale for everything that is done in the classroom, building on what the students already know, encouraging interaction and cooperation through pair and group work, etc.), awareness (drawing students’ attention to the learning process and making it explicit), self-assessment (incorporating the use of diaries and portfolios in teaching), and curricula (giving the students an overall understanding of the course outline, demands and objectives).

Nunan (1997) proposed a five-levels approach for promoting learner autonomy through making the learners aware of the goals, content and materials of teaching, involving them and letting them intervene in goal-setting procedures, allowing students to create their own goals and objectives and applying classroom content creatively in the world beyond.

Ikonen (2013) attempted to merge the principles that are common among most of the approaches discussed above into themes for the promotion of learner autonomy. These themes are choice (on learner level, learning level, curriculum level, task level, etc.), goals and needs (identifying needs, setting goals, and reflection on one’s own learning), teacher support (providing guidance and support to students), metacognition (increasing learners’ awareness about their own learning processes by making the learning process explicit to students and promoting self-evaluative practices among students by using portfolios and diaries), emotional climate (creating an atmosphere in which students feel willing and secure enough to accept the change, accepting the change and being willing to share responsibility by teachers and by expressing mutual trust and appreciation by both students and teacher), motivation (enhancing self-determined goals relate to learners’ intrinsic motivation to make them willing to take responsibility of their own learning). The boundaries between these themes, according to Ikonen (2013) are not clear-cut because they car interrelate with one another (e.g., emotional climate requires also support, metacognition, is a prerequisite in the establishment of goals and needs, choice is inherent in all the other themes) because of the multidimensionality of the concept of learner autonomy.

The frameworks elaborated above might differ in the way they view learner autonomy in that some of them such as that of Littlewood (1996) and Benson (2011) care for both in-class and out-of-class aspects of learner autonomy while others (e.g., Nunan, 1997; Lewis and Reinders, 2008; Dam, 2011; Ikonen, 2013) precisely account for in-class learner autonomy. These differences might result in some models being more comprehensive than the others and in the components of certain models becoming more efficient in promoting learner autonomy if properly executed in the FL classroom. All these frameworks, however, extremely emphasize the fact that the most important approaches for promoting learner autonomy are those strategies empowering learner choice and control over learning, increasing their awareness and metacognition, and fulfilling their learning goals, needs, and emotions. In addition, they all highly emphasize the critical role of the teacher support in this respect. Earlier studies such as that of Voller (1997), Benson (2011), Murase (2015), and Pham (2021) hypothesized that the main trait of the teacher as a facilitator is to provide psychosocial and technical support to students. The psychosocial support denotes the personal qualities of the teacher, such as being supportive, tolerant, empathic, open, motivating, and awareness promoter. The technical support, on the other side, mainly implies that the teacher helps learners to plan their learning, define their goals, find the useful materials, and evaluate their progress. These studies also acknowledged that the main issue in the relationship between teacher and learner lies in the management of power. Given that autonomous learning is chiefly based on the fact that learners take control of their own learning, their power concomitantly increased, and the teacher’s power be lessened, the teacher, who traditionally controls the classroom, has to grant some of his power to the students.

## Research Context

The concept of learner autonomy is considered to be not widely common in countries like Saudi Arabia, where teacher-centered approaches are extremely dominant. Teachers in this context are seen as authoritative figures whose main job is to give knowledge, correct learner errors, and control the whole learning. Saudi EFL students are, nonetheless, typically heavily dependent on their teachers, driven by their commands, anxious, demotivated, and disoriented. As a result, the two concepts of self-regulated and self-determined learning are yet to be recognized in the Saudi EFL contexts. Only a handful portion of studies have been conducted in the Saudi context on EFL learner autonomy. Some of such studies investigated teachers’ and students’ perceptions about this concept (e.g., Almusharraf, 2018, 2020; Alrabai, 2017a; Asiri and Shukri, 2018; Alhejaily, 2020). These investigations found that the teachers’ practices were mainly traditional (i.e., teacher-centered teaching), with EFL students perceived by participants teachers and students alike as passive, dependent, lacking initiative, and non-autonomous. While those teachers described promoting learner autonomy a desirable goal (Alonazi, 2017), they appeared less optimistic about the feasibility of this task; attributing that in the first place, to some learner-related factors such as lack of motivation and independence and low proficiency in English and to other curricular and societal constraints (Borg and Alshumaimeri, 2019) as well as to their limited experience on how to implement learner autonomy in their EFL teaching practice because they lack the knowledge and the proper training to apply it (Alonazi, 2017; Halabi, 2018). Other studies in the Saudi EFL context (e.g., Alrabai, 2017b) attempted to measure the degree of EFL autonomy among Saudi undergraduates and to assess how learners’ autonomy associate with their EFL achievement. These studies established a significant correlation between learner autonomy and English achievement of Saudi EFL learners in that learners were found to be low autonomous and low achievers of English language. A third type of research on autonomy in Saudi Arabia assessed EFL learner’s readiness for independent learning and the factors behind preventing Saudi EFL learner from being autonomous (see Al Asmari, 2013; Tamer, 2013; Alrabai, 2017c). Similar to the findings established by similar studies in other EFL contexts such as that of Çetin and Çakır (2021) in Turkey, these studies confirmed the relatively low readiness of Saudi EFL learners for self-determined learning in terms of low perceived responsibility levels, decision-making abilities, and involvement in this kind of learning. The low readiness was attributed to a variety of possible reasons such as lack of involvement in self-directed activities; high dependency on the teacher (Alrabai, 2017c), lack of teacher proper feedback, lack of training on how to develop autonomous language learning skills (Asiri and Shukri, 2020), and many other reasons.

## Rationale and Theoretical Framework

While some studies in the Saudi EFL context have tested the influence of motivational (e.g., Alrabai, 2014; Alqahtani, 2015) and anxiety reducing strategies (Alrabai, 2015), no single study in that context has ever yet attempted to explore how the teacher autonomy-supportive practices would affect learner EFL autonomy in the language class despite that literature bounds with scholarly sources recommending theoretical suggestions to promote learner autonomy in the language classrooms. Despite the valuable theoretical assumptions provided by such studies (e.g., Reeve, 2005, 2009; Benson, 2011; Dam, 2011; Ikonen, 2013; Sella, 2014; Pichugova et al., 2016; Hu and Zhang, 2017; Suharmoko, 2017; Gurbanov and Mirzayeva, 2018; Yu, 2020; Saeed, 2021) about possible approaches for promoting learner autonomy in foreign language classes, the empirical practicality of such strategies is yet to be established as none of these studies has experimentally tested their effectiveness in promoting learner autonomy in such settings. While there have been some teacher autonomy-supportive interventions in other fields of study like the ones by Cheon et al. (2012) in physical education and Lozano-Jiménez et al. (2021) in programmes other than EFL, experimental autonomy-promotion interventions in EFL/ESL classes are very rare to date. Even with the appearance of some recent attempts to utilize autonomy-supportive strategies in language classes (see, e.g., Hu and Zhang, 2017; Ramírez, 2017; Chinpakdee, 2020; Pham, 2021), these studies appeared more concerned with the general practice of teachers rather than utilizing specific strategies to promote learner autonomy. They also lacked for comparison (control) group, which made it inapplicable to compare the results of the experimental intervention in the treatment classes with that of the traditional methods of instruction followed in the control ones. In addition, all these studies were cross-sectional and correlational in nature. These research designs do not allow to capture changes in learners’ autonomy levels overtime due to experimental interventions. Further, most of these studies, with the except of Hu and Zhang (2017), lack solid theoretical bases to build on their interventions.

The present study aims to fill in the gap of previous investigations by moving from theory to practice by implementing a controlled autonomy-promotion intervention in the foreign classroom. The need for this research is very well-established and can be expected to make a significant contribution not only to second/foreign language teaching and learning in Saudi Arabia but also to second language acquisition theory more generally. The study is novel in that it follows a systematic way of research that investigates the direct relationship between the actual use of autonomy-supportive strategies and students’ autonomy in the language classes *via* an experimentally-based two-wave longitudinal design. This original new approach to the study of L2 autonomy that has not been part of previous studies elsewhere has, compared with cross-sectional studies, a greater capacity to reveal changes in learners’ autonomy levels overtime and can therefore be expected to produce substantive new findings with regard to such an elusive construct as L2 autonomy.

This intervention is theoretically based on the self-determination theory (SDT) perspective which, according to Little (2020), has identified autonomy as a basic human need and motivational drive. SDT is an influential theory of motivation that describes and explains the relation of human basic innate psychological needs to self-determined motivation and self-regulated behavior (Deci and Ryan, 1985, 2000; Ryan and Deci, 2017, 2020). These psychological needs include needs for autonomy (need to experience volition and self-endorsement in one’s behavior), competence (need to experience improvement and a sense of being effective in one’s interactions with the environment), and relatedness (need to experience warm, close, responsive, and reciprocal care within one’s relationships). In light of this theory, the better these learners’ basic needs are satisfied, the more students thrive, flourish, and display adaptive self-determined motivation and self-regulated learning behavior (Oga-Baldwin et al., 2017; Alamer and Lee, 2019; Alamer, 2021; Alamer and Almulhim, 2021); and the more these needs are frustrated, the more students suffer, flounder, and display maladaptive motivation and autonomy (Hu and Zhang, 2017; Ryan and Deci, 2017; Reeve et al., 2018). According to Alamer (2021), SDT posits that individuals can hold a variety of motivational orientations that illuminate learners’ purpose and manner of approaching, engaging (or not engaging) in, and completing L2 tasks. Intrinsic motivation which is conceptualized by Comanaru and Noels (2009, p. 34) as pursuing an “activity in the absence of a reward contingency or control” is, according to Alamer (2021), a key concept to SDT. Another key principle of SDT is goals and needs setting. Research on SDT and goals (e.g., Koestner and Hope, 2014) has confirmed the connection between goal-setting and autonomy in that being autonomous is more likely when our goals are intrinsic and intended to satisfy our basic needs. López-Íñiguez and McPherson (2020) considered as metacognition, which refers to “knowledge, awareness, and regulation of one’s thinking” (Zimmerman and Moylan, 2009, p. 299), as a major component of self-regulated learning and therefore an essential concept for enhancing learner autonomy.

To evaluate the effectiveness of the experimental autonomy-supportive teaching intervention, the present study aims to test the following research questions:

RQ1: What are the most important strategies to promote the autonomy of Saudi EFL learners?
RQ2: Will teacher autonomy-supportive teaching enhance EFL learner’s autonomy?
RQ2: How teacher autonomy-supportive teaching would causally predict learner EFL autonomy?

## Research Design

The present study involves two critical stages. The first stage was an exploratory phase conducted to identify the autonomy-promoting strategies to be utilized in the experimental classes during the second stage. Building on findings from the first phase, the second/main phase of the study featured a teacher strategy-based intervention designed to empirically establish whether implementing autonomy-promoting strategies in language classrooms would induce positive changes in learner EFL autonomy. In this phase, the study used a longitudinal pre- and post-experimental design with a control group to provide a methodologically controlled investigation into the effects of the preselected autonomy-promotion strategies that teachers implemented in an experimental group during a 12-week EFL instruction. The purpose of the pre-test was to identify any pre-experiment differences between participants (teachers and students) in the different study groups. The purpose of the post-test, however, was to identify the post-experiment changes in teacher autonomy-promoting practices and student autonomous behavior due to treatment, independent of any pre-existing group differences.

### Participants

Participant teachers in the first stage of the study were 86 EFL teachers who represented a variety of gender (44 females and 42 males), school level (32 schoolteachers and 54 university instructors), different qualifications (24 BA, 41 MA, and 21 PhD), with age between 28 and 57 years, and EFL teaching experience between 5 and 35 years.

In the second stage of the study, the study involved the participation of four teachers and 62 Saudi EFL learners from two universities located in the west and south of Saudi Arabia. The group of teachers involved in the study at this phase belongs to a variety of age range (34–42years old), EFL teaching experience (15–22years), and different qualifications (two Ph.D., and two MA holders). Besides, 62 Saudi male and female university English-major learners speaking Arabic as L1 took part in the main study. Their age ranges from 19 to 27 and their level of language competence varies from intermediate to advance level.

## Matching and Randomization

Since the experimental intervention in this study is chiefly concerned with identifying the net effects of the teacher autonomy-supportive teaching *via* utilizing autonomy strategies on learner autonomy and to establish causality among the study variables on that basis, the effect of any variables that might cause significant differences between the study groups pre to treatment must be controlled for. This entails assigning subjects in the groups in such a way that the study groups (treatment vs. control) are equivalent in all the aspects except the manipulation of the treatment. This is achieved *via* matching and randomization procedures. Learner participants in the different groups were matched across a variety of demographics including age, gender, level of study, learning experience, proficiency level, etc. Participants teachers were matched for age, gender, qualifications, teaching experience, etc. Randomization in this study is based on the statistical principle of normal distribution and was conducted through assigning participants to the study groups: both teacher and learner participants were assigned to the study groups based on their willingness to join the group they prefer (treatment or control) after they received ample information about the objectives of the study, its methodology, the experiment, the procedures, and the anticipated outcomes. A two-condition (treatment vs. control) between-subjects ANOVA was performed on all pre-test constructs and verified that the study groups were equated and that the samples of this study were well-matched (see Tables 1 and 2).

**Table 1:** Variance between the teacher groups (treatment vs. control) on pre-test classroom observation constructs.

No.	Construct	Treatment		Control		*F* (1, 2)	*p*	*η_p_* ^2^
		M	SD	M	SD			
1.	Promoting choice	3.72	0.21	3.6	0.34	0.89	0.367	0.08
2.	Teacher autonomy support	2.85	0.45	2.75	0.86	0.21	0.654	0.02
3.	Satisfying goals and needs	3.38	0.19	3.21	0.40	0.99	0.344	0.09
4.	Satisfying metacognition	2.90	0.74	2.75	0.36	0.21	0.660	0.02
5.	Promoting relatedness	3.03	0.49	2.97	0.29	0.06	0.815	0.01
6.	Promoting competence	2.72	0.34	2.64	0.36	0.17	0.689	0.02
7.	Promoting intrinsic motivation	2.80	0.30	2.75	0.27	0.11	0.747	0.01
8.	Pre-test autonomy-supportive teaching	3.06	0.19	2.95	0.16	1.18	0.304	0.11

M, mean; SD, standard deviation; *F*, variance of the group means; *p*, significance value; *η_p_*^2^, partial eta squared.

**Table 2:** Variance between the learner groups (treatment vs. control) on pre-test autonomy constructs.

No.	Construct	Treatment		Control		*F* (1, 2)	*p*	*η_p_* ^2^
		M	SD	M	SD			
1.	Control over learning	3.00	0.93	2.16	0.87	2.54	0.137	0.18
2.	Freedom of choice	2.90	0.72	1.81	0.75	1.39	0.261	0.10
3.	Intrinsic motivation	4.00	0.94	3.24	0.97	1.76	0.209	0.13
4.	Pre-test observed autonomy	3.16	0.87	2.45	0.85	1.17	0.302	0.09
5.	Perceived choice	3.61	0.96	3.75	0.81	0.36	0.552	0.01
6.	Perceived autonomy support	4.29	0.93	4.60	0.86	0.67	0.416	0.01
7.	Goals and needs satisfaction	4.16	0.82	4.23	0.84	0.04	0.840	0.00
8.	Metacognition satisfaction	4.13	0.82	4.23	0.81	0.07	0.787	0.00
9.	Perceived relatedness	4.18	0.80	4.22	0.82	0.02	0.886	0.00
10.	Perceived competence	4.70	0.75	4.67	0.71	0.03	0.874	0.00
11.	Intrinsic motivation	4.65	0.83	4.54	0.82	0.14	0.710	0.00
12.	Pre-test reported autonomy	4.24	0.91	4.32	0.89	0.09	0.765	0.00

### Instruments

Three instruments were deployed in this study. In the first stage of the study, only one instrument was used. This instrument was an online teacher self-report measure survey that was used in the identification of the most impactful autonomy-promotion strategies. It helps in selecting the appropriate strategy for optimizing EFL autonomy-supportive teaching at the main phase of the study. This instrument was developed particularly for the purpose of this study. The macro and micro strategies in this measurement were selected based on a thorough review of a vast body of related literature in the field that theoretically recognized these strategies as having the potential of supporting EFL leaner autonomy in the classroom (e.g., Reeve, 2005, 2009; Benson, 2011; Dam, 2011; Ikonen, 2013; Sella, 2014; Pichugova et al., 2016; Hu and Zhang, 2017; Suharmoko, 2017; Gurbanov and Mirzayeva, 2018; Reeve et al., 2019; and others). More practically, these strategies were selected based on the learner BPNs highlighted by SDT, which was the theoretical base of this study and the key concepts of this theory. The initial version of this instrument comprised 12 macro strategies and 123 micro strategy for promoting EFL learner autonomy. Each strategy was ranked by teachers based on its perceived importance on a five-point Likert scale ranging from 0 (not at all important) to 4 (very important).

In the second phase, two instruments were utilized. The first instrument was a classroom observation scheme that was designed specifically for this study to assess the teacher autonomy-supportive practices and students observed autonomous behaviors in real classroom setting. Variables in these two observation protocols were designed to reflect on the autonomy-supportive teaching strategies teachers were deploying during the intervention, which were selected based on the principles of the SDT; the conceptual framework of this study. The teacher autonomy-supportive teaching was rated on a five-point frequency Likert scale ranging from 1 (never) to 5 (always); while students autonomy behavior was assessed by the number of students involving in each behavior at the moment of assessment using a five-point quantity Likert scale ranging from 1 (none) to 5 (all).

The second instrument used in this phase was a questionnaire that was developed to measure the learner autonomy variables affected by teachers’ use of different autonomy strategies. Some of the items in this measurement were adopted from the Basic Psychological Need Satisfaction and Frustration Scale (BPNSFS) by Deci and Ryan (2000) to measure learner perceived autonomy, competence, and relatedness, the Learning Climate Questionnaire (LCQ) by Black and Deci (2000) to measure perceived autonomy support, Intrinsic Motivation Inventory (IMI) scale by Ryan and Deci (2002) to evaluate participants’ interest/enjoyment, perceived competence, perceived choice, effort, value/usefulness, and anxiety/tension. Items in the student self-report measure were translated from English to Arabic and were rated on a seven-point semantic differential scale ranging from (1) very untrue to (7) very true. The verified Arabic version was administrated to student participant involved in the study.

Prior to the investigation, the whole portion of instruments was piloted among a study sample of 18 EFL teachers and 34 learners. This sample was representative of the sample recruited in the main phase of the study. In light of this piloting, some additions, omissions, and amendments were made on the study measurements. For determining the instruments validity, the tools were examined by four experts of EFL. Based on their recommendation and suggestion, the instruments were revised. The results of the Cronbach Alpha reliability analysis revealed that all the instruments (1, 2, and 3) are reliable: 0.87, 0.76, and 0.93, respectively. Variables in all instruments were screed for normality of the score distributions. Data in all constructs were normally distributed. The measurements used in this study are available in the Appendix.

### Intervention Training

In this intervention study, teachers in the experimental condition participated in a three-stages comprehensive autonomy-promoting professional development training. The purpose of this training is to help teachers to become more autonomy-supportive and less controlling in their EFL classrooms by helping them to enact autonomy-supportive instructional behaviors in EFL instruction with their own students.

The training took place in the semester preceding the actual intervention (i.e., before teachers begin interacting with students and before teacher-student relationships stabilize). On day 1 of the professional development program, an information-based 3-h workshop was conducted by the researcher. This workshop started with an overview about autonomy-supportive teaching and provides empirical evidence on its benefits compared to controlling teaching. It also involves some activities to help teachers to reflect on the autonomy-supportive and controlling aspects of their own FL instruction. Teachers then were asked to complete the teaching scenario questionnaire (see Reeve et al., 2014) to understand how much they personally endorse autonomy-supportive teaching and controlling teaching.

In the second day of the training, participant teachers were provided with videotaped examples of the autonomy-supportive instructional behaviors and revolves mostly around the nine macro strategies identified as the most important strategies at the first stage such as supporting learner freedom of choice, metacognitive skills, goals and needs satisfaction, self-regulated behaviors, sense of competence, and relatedness. As to make the training practically feasible, the methods on how to apply each autonomy-supportive instructional behavior were also discussed and then followed up by autonomy-supportive teaching simulation activities using a recommended teacher script for each activity (see the Appendix for examples of these scripts). These scripts were thoroughly concerned with helping teachers to (1) transform their preexisting controlling instructional behaviors (e.g., uttering directives) into a more autonomy-supportive alternative (e.g., providing explanatory rationales), (2) enact each individual autonomy-supportive strategy, and (3) enact all individual nine autonomy-supportive instructional behaviors into a coherent interrelated and mutually implemented autonomy-supportive instructional protocol with their students. Teachers were then allowed 6weeks to integrate autonomy-supportive instruction prescribed in the training in their EFL teaching. Since training was more concerned with the development of the teacher’s overall autonomy-supportive teaching practice, teachers were encouraged to incorporate as much autonomy-supportive strategies as applicable into each EFL teaching activity without placing special emphasis on particular autonomy-supportive strategies more than the others. Typically, teachers were advised to follow Reeve (2016) methodology for autonomy-supportive teaching deployment by breaking down autonomy-supportive teaching into three critical moments within the instructional flow to allow them to implement autonomy-supportive teaching while simultaneously delivering the prescribed curriculum. The first autonomy-supportive moment is taking students’ perspectives during the pre-lesson reflective period in which the teacher plans and prepares the instructional episode such as learning objectives, learning activities, schedule of events, etc. This stage of autonomy-supportive teaching involves supporting learners to identify their goals and needs and pre-lesson preferred choices. The second set of autonomy-supportive moments is implemented next after the lesson begins, and it involves providing learners with autonomy-promotion teacher support, during-lesson choices, and metacognitive skills support. In the third stage, teachers were asked to deploy strategies like promoting student competence, intrinsic motivation, and relatedness in order to address student’s problems that might appear as the lesson unfolds such as disengagement, poor performance, negative emotions, etc.

The third stage of the training took place in the form of group discussion in which autonomy-supportive teaching in their own classrooms was assessed. In this stage, teachers share their experiences, exchange tips and strategies for particular teaching situations, report on how their students reacted, and report on the obstacles they encountered to autonomy supportive EFL teaching.

### Intervention Experience

A period of 12-week was determined for performing the experimental intervention in the academic year 2019–2020. Throughout the experimental period, teachers in the treatment group executed the experimental intervention in using seven macro strategies (promote students’ choice ability, increase students goals and needs satisfaction, increase teacher support to learner autonomy, enhance student metacognition satisfaction, enhance relatedness satisfaction, promote students competence satisfaction, promote student intrinsic motivation) following Reeve (2016) mechanism for autonomy-supportive teaching. Each macro strategy was executed using a set of micro strategies/techniques. For instance, the following micro-strategies were recommended for the teacher to use during instruction to enhance learners’ perceived choice in the language class:

1. Offer students:
  - A variety of learning tasks and activities, from which they can choose what suits them.
  - A variety of assessment tools (e.g., tests, quizzes, and assignments).
  - Tasks with different levels of difficulty, so that they can choose tasks that suit their abilities.
  - The opportunity to be involved in class management.
2. DO NOT:
  - Limit students’ choices and options.
  - Direct students’ choices.

### Procedures and Data Collection

Data in the first phase of the study was collected from EFL teachers *via* an online questionnaire to identify the most important strategies to be incorporated in EFL instruction in the treatment group in the main phase of the study for the promotion of learner EFL autonomy. Teachers who showed a willingness to participate were provided with a link to the survey. The link directs participants to an introductory section in which they were provided with comprehensive information about the different aspects of the study. At this page, potential participants were asked to either complete an online consent form to confirm their participation and then proceed to the next page or to click a link to exist the survey if they declined the option to participate. Those who gave their consent to participate were asked to proceed to the next section to begin responding to the online survey that comprised 123 strategy/technique in which respondents were asked to express their perceptions regarding the importance of each strategy. In the final section of the survey, the respondents were asked to provide some demographic information (including gender, age range, school level, qualifications, etc.).

The selection of the two participating institutions in the main phase was randomly held. The institutions were then contacted for attaining the permission from the governing body of each institution followed by the approval of English teachers, along with students’ consent to participate in the study. Prior to assignment of participants to either control or experimental groups, the researcher visited the institutions, where the study took place and thoroughly explained to potential teacher and student participants all the aspects related to the study. Six teachers expressed their willingness to take part in the study at this phase. While two teachers showed willingness to teach for the control group, the other four teachers agreed to be involved in the pre-treatment training designed for those teachers willing to implement the experiment in the treatment group. After the training, two of the four teachers showed their unwillingness to take a further role in the study. Only four remaining teachers (two in the treatment group and two in the control group) participated in the main study.

Data in the main phase was collected twice; before the experimental treatment (pre-test) and post to treatment (post-test). The pre-test was conducted in week 2 of the term after students had actually enrolled in their classes, and the post-test was finalized 2weeks before final exams to avoid end-of-term course droppings that might affect the study conduct. Data of each test were gathered from teachers and students on the same day at their institutions during normal class time. Classroom observations were conducted first. The researcher and another trained rater visited the experimental and control classrooms to observe and make independent ratings of teachers’ autonomy-supportive vs. controlling instructional behaviors and students’ autonomous vs. controlled behavior in a naturalistic learning setting. Three systematic classroom observation intervals (7min each) were conducted to record the frequency of autonomy-supportive behaviors that the teacher demonstrates, and another three observation sessions (5min each) to record the percentage of students exhibiting autonomous behaviors in the class in a successive manner. The total time of classroom observation was 45min (36min to record teacher and students’ in-class behaviors and 9min to shift between teacher and student’s observation sessions). In each observation session, each instructional behavior was scored using a bipolar format in which the controlling behavior (scored as 1) appeared on the left side of the scoring sheet while the autonomy-supportive behavior (scored as 5) appeared on the right side. Because teachers were required to enact all autonomy-supportive instructional behaviors in every single lesson to display on a comprehensive autonomy-supportive instructional practice, the mean score of each rated micro strategy was scored and then summed up with the means of the other micro strategies to get the total mean score of the macro strategy that these micro strategies/techniques represent. The interrater reliability analysis using the Kappa statistic showed that the ratings from the two observers were highly correlated Kappa=0.793 (*p*<0.001), displaying a substantial level of agreement between raters.

The questionnaire survey was administrated next by the researcher himself to students in their classes in the absence of class teachers and any other school official whom presence might affects students’ responses. Before distributing the questionnaire, the researcher thoroughly explained the objectives of the study and the way to fill in the survey. Students took around 1h to finish responding to the whole questionnaire.

### Data Analysis

Data from the survey administered to the English teachers to identify the most important autonomy-promotion strategies in phase 1 was analyzed using descriptive statistics (mean and SD).

Since phase 2 of the study was concerned mainly with the effect of the experimental intervention on the various autonomy variables and how that effect resulted in differences between the experimental and control groups, the data gathered in this phase was analyzed longitudinally – at the beginning, and end of the treatment using univariate ANOVA and analysis of covariance (ANCOVA). This is because ANOVA and ANCOVA analyses are powerful enough to disclose the net effect size of the treatment manipulated in this study. The partial Eta squared (*η_p_*^2^) was used as an estimator of the effect size of the experiment. We followed the way used in many previous studies and statistical manuals (e.g., Cheon, 1988; Kinnear and Gray, 2010) in interpreting the value of the eta squared coefficient in order to reveal the effect of the treatment: 0.01=small effect, 0.06=moderate effect, and 0.14=large effect. In addition to univariate analyses, a multiple hierarchical regression analysis has also been performed to investigate the effects of the teacher autonomy-supportive behaviors as potential predictors of learner autonomy and to statistically determine which behaviors have the most predictive power in this variable.

## Results and Discussion

This section presents a description and discussion of the main findings that were derived from the statistical analyses tailored to answer the three research questions as follows:

### The Most Important Autonomy-Supportive Strategies

In answer to RQ1, the most impactful strategies to promote the autonomy of Saudi EFL learners were identified based on participant teachers’ responses in the first stage of the study. Only strategies which attained a mean score of (three and above out of four) were considered important. As a result, only nine macro strategies that comprise 84 micro strategies were considered important to be implanted in treatment classes in the second phase. Later, teachers who participated in the intervention training recommended taking rid of two additional macro strategies and their micro strategies since they recognized these strategies as not relevant to the context and population of the study. Therefore, only seven macro and 71 micro autonomy-supportive instructional strategies were used in the treatment stage based on teachers’ perceptions of these strategies as the most applicable in Saudi EFL classes for the purpose of promoting learners’ EFL autonomy. The strategies that teachers selected at this phase coincide with the principles of the SDT, which provided a theoretical rationale for the intervention in this study. This would allow to incorporate such principles like satisfying learners’ basic psychological needs (BPNs) into EFL course education and thus boosting students’ classroom experiences of autonomy, competence, relatedness need, choice ability, metacognition satisfaction, and intrinsic motivation of learners. The strategies selected by teachers in this study are also consistent with those *t* proposed in Lewis and Reinders (2008), Dam (2011), and Ikonen (2013) theoretical frameworks in terms of promoting learners’ choice ability and control over learning as well as learner’s metacognitive skills. They are also in line with the strategies proposed about teacher support in the models of Dam (2011) and Ikonen (2013). In addition, the strategies in this study with regard to promoting learner’s goals and needs satisfaction come consistent with those proposed in the frameworks of Nunan (1997) and Ikonen (2013). Empirically, the strategies selected in the present study for satisfying learners’ BPNs of autonomy, competence, and relatedness duplicate those used in the study of Hu and Zhang (2017) in particular. This agreement with both the theoretical frameworks and empirical investigation in the field grant the autonomy-supportive strategies proposed in this study, a promising advantage in enhancing different aspects of learner EFL autonomy, which is the ultimate goal of this study. These strategies are ranked in a descending order of the mean in the Appendix.

### Manipulation Checks

The answer to RQ2 about the effectiveness of teacher autonomy-supportive teaching intervention in enhancing EFL learner’s autonomy was obtained by comparing the pre-treatment with post-treatment scores of teacher and learner autonomy variables.

#### Pre-test Findings

A two group (treatment vs. control) between-subjects ANOVA was run to reveal the variance among the study groups prior to the manipulation of the experimental treatment. As can be noticed in Table 1, the group condition factor had a null effect on all teacher pre-test variables (*p*>0.05). This indicates that no significant differences were detected between teachers in the treatment vs. control group prior to treatment with regard to their autonomy-promotion practices.

The same ANOVA test performed on the pre-test learner observed and reported data. Results indicated a non-significant main effect of the group condition factor (*p*>0.05) on all learner observed and self-reported autonomy constructs and overall pre-test autonomy prior to treatment. This emphasizes that participants learners in the control and treatment groups were not statistically different in all the aspects of their autonomy prior to treatment (see Table 2).

These insignificant differences in all teacher and learner pre-test variables between the treatment and control learner groups are an indicator of the successful and effective matching procedures that this study employed.

#### Post-test Findings

To identify teacher variables that changed in the treatment (vs. control) condition due to the experimental intervention, a two-condition (treatment vs. control)×two-time (pre vs. post) ANCOVA test was performed after specifying the pre-test scores as covariates to control for their likely effects on post-test scores. We observe that all the teacher variables that did not significantly differ between the treatment and control groups prior to the treatment (Table 1) became significantly different between the two groups after the treatment (see Table 3). This result indicates that there was a statistically significant effect of the experimental treatment on all post-test teachers’ variables despite that it had no significant effect on any of them pre to treatment. These findings suggest that utilizing autonomy-supportive EFL instruction resulted in positive changes in all of autonomy teaching behaviors of the teachers in the experimental group compared with those in the control group. This difference between teachers in the two groups could likely be attributed to the lack of intervention in the latter group. The treatment had the largest effect on teacher practices to promote learner freedom of choice ability [*F* (1, 1)=74.66, *p*<0.001, *η_p_*^2^=0.89], indicating that this variable was the most positively affected by the utilization of autonomy-supportive intervention. This validates what has been hypothesized by Ryan and Deci (2002) and Evans (2015) that, according to SDT principles, autonomy is best supported through the provision of choice to students.

**Table 3:** Variance between the teacher groups (treatment vs. control) on post-test classroom observation constructs.

No.	Construct	Treatment		Control		*F* (1, 1)	*p*	*η_p_* ^2^
		MM	SD	MM	SD			
1.	Promoting choice	4.78	0.14	3.60	0.26	74.66	0.000	0.89
2.	Autonomy support	4.35	0.21	2.84	0.45	54.69	0.000	0.86
3.	Satisfying goals and needs	4.24	0.35	3.22	0.39	19.98	0.002	0.69
4.	Satisfying metacognition	4.29	0.30	2.69	0.80	18.40	0.002	0.67
5.	Promoting relatedness	4.05	0.34	3.17	0.37	17.10	0.003	0.66
6.	Promoting competence	4.31	0.48	2.44	0.52	36.68	0.000	0.80
7.	Promoting intrinsic motivation	4.05	0.39	3.06	0.25	25.42	0.001	0.74
8.	Post-test autonomy-supportive teaching	4.30	0.14	3.00	0.33	63.44	0.000	0.88

MM, marginal mean.

The same ANCOVA test was performed to determine the effect of the condition factor on learner observed and reported post-test autonomy while no preexisting differences were identified between the learners in the study groups in terms of their pre-test autonomy (Table 2); nevertheless, this factor had a significant effect on learner’s post-test autonomy (Table 4).

**Table 4:** Variance between the learner groups (treatment vs. control) on post-test autonomy constructs.

No.	Construct	Treatment		Control		*F* (1, 1)	*p*	*η_p_* ^2^
		MM	SD	MM	SD			
1.	Control over learning	3.24	0.72	1.89	0.68	7.53	0.019	0.41
2.	Freedom of choice	4.41	0.41	2.12	0.59	28.22	0.000	0.72
3.	Intrinsic motivation	4.46	0.72	3.01	0.61	9.55	0.010	0.47
4.	Post-test observed autonomy	4.12	0.88	2.26	0.77	18.19	0.001	0.62
5.	Perceived choice	4.25	0.59	3.52	0.82	74.75	0.000	0.56
6.	Perceived autonomy support	5.09	0.47	4.07	0.88	95.10	0.000	0.62
7.	Goals and needs satisfaction	4.77	0.60	3.76	0.96	35.78	0.000	0.38
8.	Metacognition satisfaction	4.63	0.62	3.78	0.83	20.26	0.000	0.26
9.	Perceived relatedness	4.75	0.61	3.88	0.91	28.97	0.000	0.33
10.	Perceived competence	5.20	0.71	4.38	0.77	30.60	0.000	0.34
11.	Intrinsic motivation	5.30	0.51	4.06	0.81	63.54	0.000	0.52
12.	Post-test reported autonomy	4.82	0.33	3.95	0.76	308.47	0.000	0.84

The effect of treatment on learner’s post-test observed autonomy was large [*F* (1,1) =18.19, *p*<0.01, *η_p_*^2^=0.62]. It is remarkable to find learner freedom of choice, which is regard as the core component of self-determined learning has been the variable that was most positively affected by the implementation of autonomy-promoting intervention [*F* (1,1)=28.22, *p*<0.001, *η_p_*^2^=0.72]. This verifies that positive changes in learner’s autonomous behavior was going in line with positive changes in teacher’s autonomy-supportive teaching. The students’ reported perceived choice has also been largely positively affected by the experimental treatment [*F* (1,59)=74.75, *p*<0.001, *η_p_*^2^=0.56]. It seems logical to claim that the improvements in learner self-reported perceived choice is going in line with the improvements in the teacher choice promoting practices as well as to the learner observed freedom of choice in the treatment classes. This is in lines with what all the theories on the promotion of learner autonomy share that the issue of choice is a fundamental feature of autonomy-supportive teaching and that learner autonomy is all about the ability to make choices by the learner. Littlewood (1996) highlighted that the learners’ ability and willingness to make choices independently falls at the core of the notion of autonomy. In addition, Cotterall (2000) verified that at the heart of learner autonomy lies the concept of choice. She added that learners can only be autonomous if they are aware of a range of learning options and understand the consequences of choices they make. Moreover, Ciekanski (2007) emphasized that the notion of choice and control by the learners are central to the autonomous learning approach. These findings also emphasize the vital role of teachers in supporting learner autonomy through the provision of choice to learners as highlighted by Evans (2015), who acknowledged that teachers can be most supportive of student’s autonomy when offering students choices of various learning tasks.

As shown in Table 4, all the pre-test autonomy self-reported variables that were not different between the experimental and control groups before the experimental intervention became statistically significantly different after the intervention. The adjusted mean scores [marginal means (MM)] of the significantly changed variables suggest larger positive changes in favour of the experimental group rather than the control group post to treatment (e.g., perceived choice: MM=4.25, SD=0.59 for experimental group; MM=3.52, SD=0.82 for control group). The sizes of the condition effect (*η_p_*^2^) were very large for all the post-test autonomy variables and were substantially larger than the (*η_p_*^2^) for pre-test variables confirming that all learner autonomy variables were largely impacted by the autonomy-supportive teaching. The significant *F* values, which were larger in all the post-test variables, confirmed that the effect of the condition was significant. These findings emphasize that the utilization of autonomy-supportive strategies in EFL instruction resulted in positive changes in all the aspects of learner autonomous behaviors in the experimental group. This has resulted in significant differences between learners in the two groups, which could be due to the lack of intervention in the control group where no significant changes in learner autonomy were observed. The improvements in student autonomy variables due to intervention in EFL classes in this study replicate those revealed by interventions in other disciplines (e.g., Lozano-Jiménez et al., 2021).

Comparable to the teachers’ post-test findings reported in Table 3, which reveal that the post-test overall teacher autonomy-supportive practice was largely positively influenced by the intervention autonomy [*F* (1,1)=63.44, *p*<0.001, *η_p_*^2^=0.88], students’ post-test findings reported in Table 4 indicate that the largest significant positive change was in learner’s overall self-reported autonomy [*F* (1,59)=308.47, *p*<0.001, *η_p_*^2^=0.84]. The large positive changes in the teacher and learner composite variables are reflective of the positive changes took place in the individual variables that the composite variables comprise. It congruently verifies the success of our treatment design in integrating the individual instructional behaviors into a coherent overall teacher autonomy-supportive teaching practice, which has resulted in turn in the treatment been largely affected all learner autonomy variables as partial Eta squared (*η_p_*^2^) estimator values in Table 4 show.

Teacher perceived autonomy support was one of the most positively impacted variables by the experimental intervention [F (1,59)=95.10, *p*<0.001, *η_p_*^2^=0.62]. Since, in the school setting, autonomy support is mostly related to teacher behaviors, it appears that the large improvements in teacher autonomy-promoting teaching practices have resulted in better perceptions of their learners as autonomy-supportive teachers. As Ikonen (2013) stated, when students perceived their instructor as supporting their autonomy, their relative autonomy increased. He added that students with low initial autonomy in particular, like the Saudi EFL learners, seem to benefit from perceived autonomy support, since it not only increases their autonomy but also their course performance. As a result of better teacher autonomy-practices, students of teachers in the experimental group perceived their teachers as becoming significantly more autonomy supportive and less controlling and reported experiencing significantly greater perceived choice, teacher autonomy support, intrinsic motivation, and a boosted sense of competence and relatedness, as well as a greater satisfaction about their needs and goals and metacognitive skills. Students of teachers in the control group, however, reported no significant change in all autonomy variables at post-test. In addition to the fact that these findings substantiate that teachers’ provision of autonomy support has been shown to be fully capable of nurturing all three psychological needs in SDT (Standage et al., 2006), it likewise empirically validates what has been theoretically recognized by a large body of research (see, e.g., Cheon et al., 2012, 2014; Cheon and Reeve, 2013; Reeve, 2016; and many others) that students in classrooms taught by autonomy-supportive teachers, compared to students in classrooms taught by controlling teachers, experience an impressive and meaningful range of positive educational outcomes, including greater perceived competence, more need satisfaction, greater classroom engagement, positive emotionality, greater autonomous motivation (including intrinsic motivation), enhanced well-being, better academic performance, and higher learning persistence.

Learner’s intrinsic motivation was also positively impacted by the intervention [*F* (1,59)=63.54, *p*<0.001, *η_p_*^2^=0.52]. This finding validates the vast theoretical assumptions in literature about the strong connection between students’ autonomy and their sense of intrinsic motivation, which is a core component of SDT (Deci and Ryan, 1985; Noels, 2013; Alamer and Lee, 2019). This finding also confirms that autonomy-supportive environment is strongly connected with intrinsic motivation in that in autonomy supportive contexts, intrinsic motivation is maintained and enhanced, whereas in controlled contexts, intrinsic motivation is undermined (Alamer, 2021). Black and Deci (2000) maintained that individual’s intrinsic motivation tends to be sustained or enhanced in learning contexts that support learner perceived autonomy and declines in contexts that learners perceive as controlling. In addition, Noels et al. (2003) established a positive association between learners’ perceptions of autonomy-supportive teachers and their intrinsic motivation. Further, Dörnyei (2005) confirmed that, in the language learning classrooms, teachers who were autonomy supportive and non-controlling promoted students’ intrinsic and self-determined orientations.

In line with what Deci and Ryan (2016) argued while referring to the SDT, that competence and relatedness were the two most important factors contributing to learner autonomy, these two student needs have also been positively affected by our treatment. Furthermore, other variables like students goals and needs and metacognition satisfaction were also positively influenced by the treatment. This confirms what has been established by earlier research (e.g., Koestner and Hope, 2014) that successful goal-setting is more likely when students are supported by empathetic and supportive people, rather than controlling or directive ones. It is, however, noteworthy to mention that the impact of the treatment on students’ perceptions of competence, relatedness, metacognition, and goals and needs was lower than that on aforementioned variables. This might indicate that the teacher autonomy-supportive strategies designated to promote learner basic needs to be represented in their perceptions of teacher autonomy-support, perceived choice, and intrinsic motivation worked better than those strategies elected to promote learner perceived competence, satisfaction of learner relatedness, metacognitive skills, and goals and needs satisfaction. This finding could also imply a stronger association between the teacher autonomy-supportive teaching and learner perceived choice and intrinsic motivation as elaborated earlier.

### Causal Associations

In order to figure out an answer for RQ 3 about the learner variable(s) that accounted for the relationship between teacher autonomy-supportive teaching and learner autonomy, a hierarchical multiple regression analysis was run. Before conducting this analysis, the prerequisites statistical conditions of the test were met: multicollinearity (high correlations, *r*=0.9 and above, between IVs, singularity (i.e., one IV is a combination of other variable(s)), and outliers) were avoided and removed, if any. Also, preliminary analyses were conducted to ensure no violation of the assumptions of normality, linearity, and homoscedasticity. The intercorrelations between the multiple regression variables are reported in Table 5, and the regression statistics are in Table 6.

**Table 5:** Reliability and correlations for learner autonomy variables and teacher-autonomy supportive teaching.

Variable	*α*	*Correlation matrix*								
		1	2	3	4	5	6	7	8	9
1. Perceived choice	0.86	1								
2. Perceived Autonomy support	0.83	0.206	1							
3. Goals and needs satisfaction	0.72	0.260	0.014	1						
4. Metacognition satisfaction	0.77	0.532^**^	0.043	0.597^**^	1					
5. Perceived relatedness	0.85	0.371^*^	0.440^*^	0.209	0.409^*^	1				
6. Perceived competence	0.93	0.470^**^	0.231	0.408^*^	0.621^**^	0.646^**^	1			
7. Intrinsic motivation	0.91	0.599^**^	0.495^**^	0.335	0.578^**^	0.693^**^	0.617^**^	1		
8. Learner autonomy	0.87	0.713^**^	0.446^*^	0.364^*^	0.593^**^	0.782^**^	0.771^**^	0.887^**^	1	
9. Teacher autonomy-supportive teaching	0.77	0.579^**^	0.561^**^	0.294	0.266	0.414^*^	0.367^*^	0.466^**^	0.583^**^	1

*a*, reliability coefficient.

* Statistical significance: *p*<0.05;

** Statistical significance: *p*<0.01.

**Table 6:** Hierarchical regression model of EFL learner autonomy.

	*R*	*R* ^2^	∆*R*^2^	*B*	*SE*	*β*	*t*
Step 1	0.58	0.34^**^	_____				
Teacher autonomy-supportive teaching				3.20	0.08	0.583^**^	3.79
Step 2	0.96	0.93^***^	0.586				
Teacher autonomy-supportive teaching				0.090	0.07	0.016	0.207
Choice				0.332	0.07	0.386^***^	4.56
Autonomy support				0.147	0.06	0.251^*^	2.51
Goals and needs				0.004	0.06	0.005	0.063
Metacognition				0.011	0.04	0.018	0.245
Relatedness				0.007	0.05	0.011	0.123
Competence				0.293	0.07	0.366^**^	3.94
Intrinsic motivation				0.170	0.08	0.220^*^	2.28

*R*^2^, amount of variance explained by IVs; ∆*R*^2^ (*R*^2^Change), additional variance in DV; *F*, variance of the variables means; *B*, unstandardized Beta coefficient; *β*, standardized Beta coefficient (values for each variable are converted to the same scale so they can be compared); *SE*, standard error; *t*, estimated coefficient (B) divided by its own SE. If *t*<2 the IV does not belong to the model.

* *p*<0.05;

** *p*<0.01;

*** *p*<0.001.

A two-step hierarchical multiple regression was conducted to investigate the predictive power of the teacher autonomy teaching as independent variable (IV) to predict learner overall post-treatment autonomy as a dependent variable (DV) in the treatment group, after controlling for the effect of learner perceived autonomy variables as mediators (MVs). In the first step of the regression, one predictor (teacher autonomy-supportive teaching) was entered. This model was statistically significant [*F* (1, 28)=14.39; *p*<0.001] and explained 34% of variance in learner post-test overall autonomy after controlling for the effect of learner variables. After entry of learner variables (six predictors): perceived choice, goals and needs, metacognition, relatedness, competence, and intrinsic motivation at Step 2, the total variance explained by the model as a whole was 93% [*F* (8, 21)=32.59; *p*<0.001]. The introduction of learner variables explained additional 59% of variance in learner post-test overall autonomy, after controlling for the effect of teacher autonomy-supportive teaching [∆*R*^2^=0.59; *F* (7, 21)=23.59; *p*<0.001]. In Step 2, the effect of teacher autonomy-supportive teaching style, which was significant in the first step, did not add significantly to the explained variance in the model (*p*=0.838, *β*=0.016). This means that the association between the IV (teacher autonomy-supportive teaching) and DV (learner autonomy) is completely accounted for by the MVs (learner autonomy variables). In the final adjusted model, four out of eight predictor variables were statistically significant, with learner perceived choice recording the highest Beta value (*β*=0.386, *p*<0.001) and thus being the strongest predictor of post-treatment learner autonomy, followed by learner perceived competence (*β*=0.366, *p*<0.01), perceived teacher support (*β*=0.251, *p*<0.05) and learner intrinsic motivation (*β*=0.220, *p*<0.05), respectively. Learner perceptions of their goals and needs, metacognition, and relatedness satisfaction did not, however, make significant unique contributions to the model (see Table 6).

The findings revealed by the regression analysis verify those reported by the other analyses in that teacher autonomy-supportive teaching significantly and positively affected all aspects of learner autonomy (both observed and self-reported). These findings are also comparable in learner perceived choice, autonomy support, intrinsic motivation, competence were the variables that were mostly impacted by the utilization of the autonomy-promoting strategies and, at the same time, the variables significantly predicting learner autonomy in the causal model in contrast to the other variables (goals and needs, metacognition, and relatedness), which has been found to be less impacted by the intervention and consequently not significantly adding to the model.

The experimentally-based and longitudinally-designed nature of this study, the well-matched sample, the careful experiment execution procedures, and the rigorous statistical analyses conducted to identify the study findings made us confident that the results this study came up with are not accidental but rather due to experimental intervention. These results empirically validate the theoretical assumptions about the practicality of autonomy-supportive teaching in promoting EFL learner autonomy (e.g., Reeve, 2005, 2009; Benson, 2011; Dam, 2011; Ikonen, 2013; Sella, 2014; Pichugova et al., 2016; Hu and Zhang, 2017; Suharmoko, 2017; Gurbanov and Mirzayeva, 2018; Reeve et al., 2019; Yu, 2020; Saeed, 2021). They also verify the fact that pedagogical autonomy interventions in foreign language classroom setting are not only possible as theoretically hypothesized but also actually effective as is in other fields (e.g., Cheon et al., 2012; Lozano-Jiménez et al., 2021). Further, the study findings experimentally support the theoretical claims about the feasibility, desirability, and teachability of autonomy in classroom setting (see, e.g., Ellis and Sinclair, 1989; Ikonen, 2013; Raya et al., 2020; Reeve, 1998), and the applicability of this concept to be incorporated in L2 instruction across different EFL contexts even if it has not been a part of the educational background in that context like in Saudi Arabia. Further, the study empirically acknowledged the significant role of the teacher as a facilitator in providing psychosocial and technical support to promote student autonomy (see Voller, 1997; Benson, 2011; Murase, 2015; Lozano-Jiménez et al., 2021; Pham, 2021).

## Conclusion, Contributions, Implications, and Limitations

This controlled experimental study attempted to test the effect of teacher autonomy-supportive teaching on learner EFL autonomy by manipulating an experimental intervention in English language classes using autonomy-promotion strategies. The intervention was manipulated in two treatment groups for 12weeks. The findings of the study revealed significant positive changes in all teacher’s and learner’s post-treatment autonomy variables in the treatment group due to the experimental intervention in this group. No significant positive changes were recorded for any of the teacher and learner autonomy variables in the control group, most likely to the lack of intervention in that group. In addition, the relationship between the teacher autonomy-supportive teaching and learner autonomy has been found to be causally mediated by learner perceived choice, competence, teacher support, and intrinsic motivation with learner perceived choice being the strongest predictor of learner autonomy. The major contribution of this study is that it is the first in the Saudi EFL setting, and might be elsewhere in the world, to empirically validate the actual practicality of autonomy-supportive strategies in promoting learner’s EFL autonomy by manipulating a controlled longitudinal experimental intervention in actual classroom setting. It therefore substantiates the theoretical assumptions provided by many theoretical frameworks in the field about the effectiveness of such strategies. The study is also the first to experimentally assess the causal relationship between the teacher autonomy teaching and learner autonomy as well as between learner overall autonomy and its underlying composite constructs (e.g., *learner perceived choice*, which this study has empirically validated as the strongest predictor of learner autonomy in language class) and to emphasize the crucial role of the teacher as a facilitator in promoting significant aspects of EFL learner autonomy based on experiment-driven evidence.

The recommendations provided here concern EFL teachers and education authorities. The role of the teacher needs to change from someone who is controlling or demanding to be a facilitator who expresses trust in the students’ abilities and guides and helps them in achieving their learning goals. Students should be empowered and released from the teacher control, granted a larger space of freedom of choice, more control over learning, and more involvement in decision-making process. The teacher should thoroughly care for and seriously attempt to satisfy the BPNs of the learner in terms of their needs of autonomy, competence, relatedness, intrinsic motivation, and metacognitive skills of need identification, goal-setting, learning planning, monitoring, and assessment.

Education authorities should take wherever necessary steps to maximize learner autonomy in EFL class. One vital way is to build the EFL curriculum around learner autonomy by integrating learning tasks and activities that take learner needs, goals, interests, and all aspects of learner autonomy into account. In addition, controlling institutional norms are to be minimized in order to allow for learner independence to develop. One possible way to do that is to allow students to have a say in EFL curriculum design and in choosing what to learn in the EFL class. Moreover, EFL teachers and students alike should be involved in training programs that equip them with the principles of learner autonomy and the ways this concept can be put into action in language classes.

One limitation of this study pertains to the fact that there is no single best strategy for promoting EFL learner autonomy and that there are no rock-solid rules in this regard due to the differences among EFL learners in different contexts in their social, educational and cultural backgrounds, age, proficiency level, etc. Based on this, there is still a lot of space available for the investigation and validation of more strategies with learners in other EFL/ESL contexts around the globe beyond the ones tested in the current investigation. In addition, all student participants in this study were adult university students. Future research endeavours could involve learners of younger age (e.g., school students) to assess the effectiveness of such interventions in promoting their EFL autonomy. A third limitation of this study is that it did not investigate the effectiveness of autonomy-supporting strategies on learners’ EFL achievement. Thus, future research efforts should examine how the utilization of such strategies would affect actual learner achievement. This could be taken forward by identifying practical means by which to utilize autonomy-promoting strategies in classroom settings and to evaluate their effects on learners’ autonomy as a first step, and on their actual achievement as a subsequent step. If it turns out that the implementation of autonomy-promoting strategies in the language classroom has resulted not only in higher autonomy in learners, but also leads to better EFL achievement, then the implications of such a study would be very far-reaching indeed.

## Data Availability Statement

The original contributions presented in the study are included in the article/Supplementary Material, further inquiries can be directed to the corresponding author.

## Ethics Statement

Ethical review and approval was not required for the study on human participants in accordance with the local legislation and institutional requirements. The patients/participants provided their written informed consent to participate in this study.

## Author Contributions

The author confirms being the sole contributor of this work and has approved it for publication.

## Conflict of Interest

The author declares that the research was conducted in the absence of any commercial or financial relationships that could be construed as a potential conflict of interest.

## Publisher’s Note

All claims expressed in this article are solely those of the authors and do not necessarily represent those of their affiliated organizations, or those of the publisher, the editors and the reviewers. Any product that may be evaluated in this article, or claim that may be made by its manufacturer, is not guaranteed or endorsed by the publisher.
