# Supplementary material for: The Influence of Autonomy-Supportive Teaching on EFL Students’ Classroom Autonomy: An Experimental Intervention
Source: Front Psychol. 2021 Sep 8;12:728657. doi: 10.3389/fpsyg.2021.728657 (PMC8455827; doi:10.3389/fpsyg.2021.728657)
Supplement: Supplementary file 1 [file Data_Sheet_1.docx]

**Appendix**

**1. Autonomy-Supportive Teaching Strategies**

| 1. **Promote student Choice ability** | M | SD |
| --- | --- | --- |
| Allow students to: | | |
| 1. take part in deciding the course schedules. | 3.63 | .63 |
| 2. take part in decision-making. | 3.33 | .71 |
| 3. choose their own learning strategies. | 3.21 | .56 |
| 4. choose learning materials that suit them. | 3.18 | .49 |
| 5. approach tasks in their personal ways. | 3.11 | .58 |
| 6. be involved in class management. | 3.02 | .55 |
| Offer students: | | |
| 7. a variety of assessment tools (e.g. tests, quizzes, assignments). | 3.71 | .54 |
| 8. a variety of learning tasks and activities. | 3.65 | .59 |
| 9. tasks with different levels of difficulty, so that they can choose tasks that suit their abilities. | 3.58 | .47 |
| DO NOT: | | |
| 10. limit students’ choices and options. | 3.54 | .68 |
| 11. direct students’ choices. | 3.43 | .51 |
| 2. **Increase student Goals and Needs satisfaction** | | |
| 12. Support and guide students to achieve their learning goals. | 3.73 | .63 |
| 13. Help students in setting their learning goals. | 3.64 | .72 |
| 14. Help students identify their learning needs. | 3.62 | .69 |
| 15. Try to adjust your teaching method to suit students learning goals. | 3.46 | .56 |
| 16. Allow students to work according to their learning goals. | 3.29 | .66 |
| 17. Try to adjust the course content to suit students learning goals. | 3.27 | .72 |
| 3. **Increase Teacher Support to learner autonomy** | | |
| 18. Listen carefully and pay attentions to students. | 3.88 | .51 |
| 19. Answer students’ questions fully and carefully | 3.86 | .49 |
| 20. Offer help when students need it. | 3.83 | .81 |
| 21. Encourage students to ask for help and advice. | 3.72 | .77 |
| 22. Be thorough and intelligible when helping students. | 3.70 | .69 |
| 23. Create a pleasant and supportive classroom atmosphere. | 3.68 | .75 |
| 24. Avoid negatively evaluating student’s poor performance through criticism or rigid messages and rather treat students’ poor performances as problems to be solved. | 3.67 | .81 |
| 25. Rely on informational, flexible, and noncontrolling language that convey flexibility and minimize pressuring statements. | 3.63 | .84 |
| 26. Encourage students to help each other. | 3.59 | .65 |
| 27. Encourage students to ask questions and give suggestions and comments. | 3.54 | .71 |
| 28. Allow students to work with a partner/in groups. | 3.52 | .54 |
| 29. Help students to diagnose the underlying causes of their poor performance and take the action needed to address the problem. | 3.45 | .78 |
| 30. adjust the tasks’ level of difficulty to students’ ability level. | 3.42 | .52 |
| 31. Acknowledge and accept students’ expressions of negative affect and resistance and initiate discussions with learners about the causes of these feeling and how to overcome them. | 3.31 | .79 |
| DON NOT: | | |
| 32. utter directives, commands, compliance requests, and controlling questions. | 3.56 | .56 |
| 33. undermine students’ thoughts, feelings, and actions. | 3.52 | .48 |
| 34. use external regulators such as incentives, rewards, and fixed deadlines. | 3.49 | .52 |
| 4. **Enhance student Metacognition satisfaction** | | |
| 35. Help learners correct their learning errors and mistakes. | 3.67 | .54 |
| 36. Provide explanatory rationales that explains the use, value, and importance of doing each learning activity. | 3.62 | .58 |
| 37. Help students identify their strengths and weaknesses. | 3.60 | .62 |
| 38. Offer students opportunities to work to develop their weaknesses. | 3.55 | .77 |
| 39. Discuss learning objectives with students. | 3.54 | .44 |
| 40. Discuss learning outcomes with students. | 3.51 | .49 |
| 41. Offer guidance to students in the evaluation of their learning process. | 3.49 | .66 |
| 42. Provide students with informative feedback. | 3.48 | .62 |
| 43. Allow students to self-assess their progress. | 3.44 | .74 |
| 44. Allow students to learn according to the learning styles that suit me best. | 3.39 | .79 |
| 45. Provide students with tools such as portfolios and learning diaries to evaluate their learning process. | 3.34 | .63 |
| 5. **Enhance student Relatedness satisfaction** | | |
| 46. Accepts every student the way he/she is. | 3.84 | .67 |
| 47. Be considerate and compassionate. | 3.81 | .51 |
| 48. Show warmth to students. | 3.79 | .44 |
| 49. Allow students to easily express their feelings to you. | 3.73 | .62 |
| 50. Allow students to interact with him. | 3.71 | .48 |
| 51. Show care and concerns about students’ progress. | 3.70 | .42 |
| 52. Allow students to be open towards you. | 3.69 | .58 |
| 53. Shows interest in students’ thoughts, suggestions, and viewpoints. | 3.55 | .46 |
| 54. Allow students to question his/her practices that do not work well in my opinion. | 3.52 | .70 |
| 6. **Promote student Competence satisfaction** | | |
| 55. Encourage students to engage in classroom discussions. | 3.68 | .52 |
| 56. Show students you believe in their abilities. | 3.67 | .50 |
| 57. Encourage students to pay much effort and persistence to achieve their goals. | 3.62 | .77 |
| 58. Encourage students to believe in their abilities. | 3.61 | .62 |
| 59. Deal properly with learner’s unrealistic beliefs associated with learning English. | 3.59 | .81 |
| 60. Help students to feel good about themselves when learning English. | 3.58 | .72 |
| 61. Make learning appealing and challenging. | 3.51 | .76 |
| 62. Stimulate students to do difficult tasks that require innovation on their part. | 3.46 | .79 |
| 7. **Promote student Intrinsic Motivation** | | |
| 63. Help students understand that learning English is much beneficial far more than just passing exams. | 3.82 | .76 |
| 64. Praise students and celebrate their improvements and mastery. | 3.79 | .57 |
| 65. Make learning English enjoyable. | 3.77 | .47 |
| 66. Controls students’ feelings of anxiety in language class. | 3.75 | .51 |
| 67. Inspire students to learn English. | 3.71 | .55 |
| 68. Spark students’ interest, enjoyment, curiosity, and enthusiasm. | 3.69 | .81 |
| 69. Help students in finding out what motivates them in learning English. | 3.65 | .73 |
| 70. Connect English learning with student’s life outside class. | 3.63 | .62 |
| 71. Build instructional activities around students’ interest, goals, and needs. | 3.39 | .69 |

**2. Teacher Classroom Observation Scale**

| Variable | Behavior | Frequency | | | | |
| --- | --- | --- | --- | --- | --- | --- |
| **Promoting choice** |  | Never | Rarely | Occasionally | Often | Always |
|  | Allows students to choose learning strategies. |  |  |  |  |  |
|  | Allows students to choose learning materials. |  |  |  |  |  |
|  | Uses varied learning tasks and activities. |  |  |  |  |  |
|  | Uses varied assessment tools. |  |  |  |  |  |
|  | Offers tasks with different levels of difficulty. |  |  |  |  |  |
|  | Limits students’ choices and options. |  |  |  |  |  |
|  | Directs students’ choices. |  |  |  |  |  |
| **Satisfying goals and needs** | helps students identify their learning needs and goals. |  |  |  |  |  |
|  | allows students to work according to their learning goals. |  |  |  |  |  |
|  | adjusts teaching method to suit students goals. |  |  |  |  |  |
| **Teacher autonomy support** | Involves students in decision-making. |  |  |  |  |  |
|  | offers help when students need it. |  |  |  |  |  |
|  | answers students’ questions fully and carefully |  |  |  |  |  |
|  | listens carefully and pays attentions to students. |  |  |  |  |  |
|  | encourages students to ask questions and give suggestions. |  |  |  |  |  |
|  | Undermines students’ thoughts, feelings, and actions. |  |  |  |  |  |
|  | Utter directives and commands. |  |  |  |  |  |
|  | Uses controlling questions and statements. |  |  |  |  |  |
| **Satisfying Metacognition** | Discusses learning objectives with students. |  |  |  |  |  |
|  | Discusses learning outcomes with students. |  |  |  |  |  |
|  | Provide explanatory rationales. |  |  |  |  |  |
|  | allows students for self-assessment. |  |  |  |  |  |
|  | provides students with informative feedback. |  |  |  |  |  |
| **Promoting Relatedness** |  | | | | | |
|  | allows students express their feelings to him/her. |  |  |  |  |  |
|  | shows interest in students’ thoughts. |  |  |  |  |  |
|  | allows students to interact with him. |  |  |  |  |  |
|  | shows care and concerns about students’ progress. |  |  |  |  |  |
|  | is considerate. |  |  |  |  |  |
| **Promoting competence** | Believes in students’ abilities. |  |  |  |  |  |
|  | Encourages students to engage in classroom discussions. |  |  |  |  |  |
|  | Makes learning appealing and challenging. |  |  |  |  |  |
|  | Inspires students to do difficult tasks. |  |  |  |  |  |
|  | Deal properly with students’ unrealistic beliefs. |  |  |  |  |  |
|  | Encourages students to pay much effort and persistence. |  |  |  |  |  |
| **Promoting intrinsic motivation** |  |  |  |  |  |  |
|  | makes learning English enjoyable. |  |  |  |  |  |
|  | inspires students to learn English. |  |  |  |  |  |
|  | helps students value learning English |  |  |  |  |  |
|  | uses topics that are relevant to students’ life. |  |  |  |  |  |
|  | controls students’ anxiety in class. |  |  |  |  |  |
|  | praises and celebrates students’ mastery. |  |  |  |  |  |

3. **Student Classroom Observation Scale**

| Variable | Behavior | Quantity | | | | |
| --- | --- | --- | --- | --- | --- | --- |
| **Freedom of choice** |  | None | Few | Some | Most | All |
|  | students choose learning strategies. |  |  |  |  |  |
|  | students choose learning materials. |  |  |  |  |  |
|  | Students use varied learning tasks. |  |  |  |  |  |
|  | Students use varied assessment tools. |  |  |  |  |  |
|  | students’ have different choices and options. |  |  |  |  |  |
|  | students direct their choices. |  |  |  |  |  |
| **Control over learning** |  | | | | | |
|  | students decide the course schedules. |  |  |  |  |  |
|  | Students freely express their thoughts and feelings. |  |  |  |  |  |
|  | Students aware of their learning needs and goals. |  |  |  |  |  |
|  | Students aware of learning objectives. |  |  |  |  |  |
|  | Students aware of learning outcomes. |  |  |  |  |  |
|  | students aware of their strengths and weaknesses. |  |  |  |  |  |
|  | students involved in class management. |  |  |  |  |  |
|  | students self-assess their progress. |  |  |  |  |  |
|  | students interact with the teacher. |  |  |  |  |  |
|  | Students show confidence in their ability. |  |  |  |  |  |
|  | students engage in classroom discussions. |  |  |  |  |  |
|  | students do difficult tasks. |  |  |  |  |  |
| **Intrinsic motivation** | students pay much effort to learn English. |  |  |  |  |  |
|  | students feel interested to learn English. |  |  |  |  |  |
|  | students feel enthused to learn English. |  |  |  |  |  |
|  | Students feel inspired to learn English. |  |  |  |  |  |
|  | Students enjoy learning English. |  |  |  |  |  |
|  | Students feel anxious in language class. |  |  |  |  |  |

**4.** **Student Questionnaire**

**1. Perceived choice**

*In my English class this semester****, ……………***

1. I feel a sense of choice and freedom in the things I do.

2. I feel that my decisions reflect what I really want.

3. My daily activities feel like a chain of obligations. (R)

4. I feel my choices express who I really am.

5. I do what I do because it interests me.

6. I do what I do because I have to. (R)

7. I feel pretty free to do whatever I choose to.

8. What I do is often not what I'd choose to do. (R)

9. My teacher directs my choices too much. (R)

**2. Perceived Autonomy Support**

*In my English instructor this semester****, ……………***

1. I feel understood by my instructor.

2. I am able to be open with my instructor during class.

3. My instructor conveyed confidence in my ability to do well in the course.

4. I feel that my instructor accepts me.

5. My instructor encourages me to ask questions.

6. My instructor answers my questions fully and carefully.

7. My instructor listens to how I would like to do things.

8. My instructor handles people's emotions very well.

9. I don't feel very good about the way my instructor talks to me.

10. My instructor allows me to question his/her practices that do not work well in my opinion.

11. My instructor offers help when I need it.

12. My instructor provides me with helpful feedback.

13. My instructor shows interest in my thoughts, suggestions, and viewpoints.

14. My instructor shows care about my progress.

15. My instructor helps me to properly deal with unrealistic beliefs associated with learning English.

16. My instructor helps me connect what I learn in English class with my life outside class.

17. My instructor encourages me to engage in classroom discussions.

18. My instructor praises me and celebrates my accomplishments.

19. My instructor helps me to feel good about myself when learning English.

20. My instructor involves me in decision-making.

**3. Goals and needs satisfaction**

*Thinking of my English language teacher this semester, he/she ………………….*

1. helps me understand the goals of the course.

2. helps me understand what I need to do to accomplish the goals of the course.

3. helps me identify my learning needs.

4. helps me in setting my personal learning goals.

5. attempts to adjust the course content to suit my learning goals.

6. attempts to adjust his/her teaching method to suit my learning goals.

7. allows me to work according to my learning goals even if they do not match my English teacher’s goals

**4. Metacognition satisfaction**

*Thinking of my English language teacher this semester, he/she ………………….*

1. helps me to choose learning strategies that suit me best.

2. allows me to learn according to the learning styles that suit me best.

3. helps me identify my strengths and weaknesses.

4. allows me to evaluate my process myself.

5. offers guidance in the evaluation of the learning process.

**5. Relatedness satisfaction**

*Thinking of my English language teacher this semester, ………………….*

1. I feel that my teacher cares about me as a person.

2. I feel close and connected with my teacher.

3. I have the impression that my teacher dislikes me. (R)

4. I experience a warm feeling with my teacher.

5. I feel like I could really trust my teacher.

6. I don’t feel like I could really trust my teacher. (R)

7. I feel that my teacher is cold and distant towards me. (R)

8. I’d like a chance to interact with my teacher more often.

9. It is likely that my teacher and I could become friends.

10. I really doubt that my teacher and I would ever be friends. (R)

11. I’d really prefer not to interact with my teacher in the future. (R)

**6. Competence satisfaction**

*In my English class this semester****, ……………***

1. I feel confident that I can do things well.

2. I feel capable at what I do.

3. I feel insecure about my abilities. (R)

4. I have serious doubts about whether I can do things well. (R)

5. I feel competent to achieve my goals.

6. I feel I can successfully complete difficult tasks.

7. I feel disappointed with many of my performance. (R)

8. I feel like a failure because of the mistakes I make. (R)

9. I think I did pretty well at learning English, compared to other students.

10. I am satisfied with my performance at learning English.

**7. Intrinsic Motivation**

*Thinking of learning English language this semester, ………………….*

1. Learning English is fun to do.

2. Learning English was a boring activity. (R)

3. Learning English did not hold my attention at all. (R)

4. learning English is a challenge that I enjoy.

*Thinking of myself this semester, ………………………………. .*

5. I put a lot of effort into learning English.

6. It is important to me to do well at learning English.

7. I do not try very hard to do well at learning English. (R)

8. I believe learning English could be of some value to me.

9. I do not feel nervous at all while learning English. (R)

10. I feel very tense while learning English.
